# Psilocybin-assisted psychotherapy for treatment-resistant obsessive–compulsive disorder: protocol for an open-label pilot study

**DOI:** 10.1192/bjo.2025.10895

**Published:** 2025-12-15

**Authors:** Nicole Ledwos, Jenna Baer, Muhammad Ishrat Husain, Daniel M. Blumberger, Rachel Patterson, Elizabeth Hollingdrake, Ezmond Cheung, Colin Hawco, Christoph Zrenner, Brigitte Zrenner, Jamie D. Feusner, Susan L. Rossell, David J. Castle, Gwyneth Zai

**Affiliations:** General Adult Psychiatry and Health Systems Division, Centre for Addiction and Mental Health, Toronto, Ontario, Canada; Department of Psychiatry, Temerty Faculty of Medicine, University of Toronto, Toronto, Ontario, Canada; Campbell Family Mental Health Research Institute, Centre for Addiction and Mental Health, Toronto, Ontario, Canada; Institute of Medical Science, Temerty Faculty of Medicine, University of Toronto, Toronto, Ontario, Canada; Temerty Centre for Therapeutic Brain Intervention, Centre for Addiction and Mental Health, Toronto, Ontario, Canada; Brain Health Imaging Centre, Centre for Addiction and Mental Health, Toronto, Ontario, Canada; University Psychiatry Hospital, University of Tübingen, Tübingen, Germany; Centre for Mental Health, Swinburne University, Melbourne, Victoria, Australia; Department of Women’s and Children’s Health, Karolinska Institutet, Stockholm, Sweden; Centre for Mental Health Service Innovation, University of Tasmania, Hobart, Tasmania, Australia

**Keywords:** OCD, treatment-resistant OCD (TR-OCD), psilocybin therapy, clinical trial, protocol

## Abstract

**Background:**

Obsessive–compulsive disorder (OCD) is a debilitating mental disorder commonly treated with selective serotonin reuptake inhibitors, atypical antipsychotic augmentation and cognitive–behavioural therapy. However, up to 60% of people with OCD do not respond to these treatments. Therefore, a novel intervention, psilocybin-assisted psychotherapy (PAP), is an option of interest. Moreover, there is a need to better understand the mechanisms underpinning PAP’s effect on OCD symptoms.

**Aims:**

We aimed to (a) establish the feasibility, tolerability and safety of administering PAP to adults with treatment-resistant OCD; (b) provide preliminary data on the clinical effects of PAP for treatment-resistant OCD, to inform the design of larger clinical trials; and (c) compare neuroimaging and neurophysiological markers pre- and post-PAP in treatment-resistant OCD.

**Method:**

In this 12-week open-label trial, ten adults with treatment-resistant OCD will receive one 25 mg dose of psilocybin combined with psychological support. Feasibility, tolerability and safety will be assessed throughout. Clinical outcomes will be measured with the Yale–Brown Obsessive–Compulsive Scale. Exploratory measures will include brain imaging examining changes in dynamic connectivity from pre to post treatment, electroencephalogram to investigate changes in brain dynamics associated with psilocybin under acute conditions, and transcranial magnetic stimulation-electroencephalogram measures between baseline, provocation of OCD symptoms and up to 1-week post-dose.

**Results:**

The study will provide important preliminary data on the feasibility and efficacy of PAP in adults with treatment-resistant OCD, as well as inform our understanding of neurobiological mechanisms.

**Conclusions:**

The findings of the study will inform the design of larger randomised controlled trials and advance the field of psychedelic-assisted therapies.

Obsessive–compulsive disorder (OCD) is characterised by intrusive thoughts, images or urges, which are typically accompanied by repetitive behaviours or mental acts.^1^ OCD is a highly debilitating disorder, with patients typically experiencing severely impaired psychosocial functioning and quality of life.^2^ Onset of OCD commonly occurs during early adolescence.^3^ Current treatment options for OCD include pharmacotherapy with selective serotonin reuptake inhibitors (SSRIs) or SSRIs augmented with atypical antipsychotics.^4^ In addition, cognitive–behavioural therapy and exposure and response prevention therapy have been moderately successful. However, up to 60% of patients with OCD fail to respond to available treatment options.^5,6^ For cases of refractory OCD, novel approaches using repetitive transcranial magnetic stimulation (TMS) have shown some efficacy for some patients, whereas bilateral deep-brain stimulation has shown promise as an invasive treatment of last resort.^4^ Given the considerable burden of OCD on patients, and because many do not respond to first-line treatments, there is an urgent need for novel and effective interventions.

Psychedelic therapy has emerged as a promising intervention for several mental and substance use disorders, including OCD. The term ‘classic psychedelic’ encompasses a broad category of drugs that act as full or partial agonists of the serotonin 2A receptor (5-HT2A). This group of agents includes psilocybin, lysergic acid diethylamide, mescaline, ayahuasca and N,N-dimethyltryptamine. Research investigating the therapeutic potential of classic psychedelics for a range of mental health and addictive disorders was conducted in the mid-1900s.^7^ However, the Controlled Substances Act in the USA and similar legislation in other counties resulted in an embargo on psychedelic research.^8^ In the past 20 years, there has been a resurgence in research investigating the clinical effects of classic psychedelics.^9^ Most notably, psilocybin-assisted psychotherapy (PAP) has been gaining significant traction as a promising intervention for mental disorders, including end-of-life distress and treatment-resistant depression. Recent studies have suggested a shift in terminology from PAP to psilocybin-assisted therapy. However, as our protocol was approved before these changes, we have chosen to retain the use of PAP for consistency and alignment with the approved protocol. Although some have suggested the primary role of the therapist is to ensure the psychological and physical safety of the participant,^10^ psychotherapy has nevertheless been shown to play an important role in facilitating treatment outcome.^11,12^

Psilocybin is a chemical compound that naturally occurs in certain species of mushroom. It is a tryptamine alkaloid, which is chemically similar to the neurotransmitter serotonin and the essential amino acid, tryptophan. Psilocybin readily crosses the blood-brain barrier, and is as an agonist of the brain’s 5-HT2a receptors.^13^ Typical effects include altered states of consciousness, experienced through visual effects, changes in perception, distortions of time, a sense of awe, novel perspectives and personal insight.

## The effect of psilocybin on the brain system

Recent research has focused on the impact that psilocybin has on the default mode network (DMN). The DMN comprises several brain regions, including the medial pre-frontal cortex, portions of the precuneus/posterior cingulate cortex and the angular gyrus.^14^ Together, these structures form an integrated network that is involved in self-referential thinking, memory and moral judgements. There is a growing body of literature to suggest that those with OCD and OCD-related disorders, such as body dysmorphic disorder, may have abnormal activation and/or connectivity patterns within the DMN and between other networks such as the salience network and dorsal attention network.^15–17^ Therefore, these findings suggest a role for psychedelics in people with OCD. Indeed, recent work investigating neural mechanisms in patients with OCD have found less deactivation in two anterior nodes of the DMN (medial and superior frontal cortex) compared with healthy controls, when transitioning between a rest condition and a pleasant emotional condition.^16^ Although a study suggested that psilocybin may reduce functional connectivity within the DMN alongside increasing connectivity to other brain networks.^18^ Decreased blood flow within the DMN nodes are correlated with neuronal desynchronisation, which has been hypothesised to result in greater mental flexibility and reduced self-referential thinking.^19^ Thus, it has been postulated that psilocybin can help the brain to ‘reset’ maladaptive networks, and that this has particular relevance for OCD.

## Current literature on psilocybin for OCD

To date, there are several case reports indicating psilocybin-mediated relief of OCD symptoms (*N* = 5).^20–24^ The most recent report of a 33-year-old male with debilitating OCD symptoms and a history of major depressive disorder, Tourette syndrome and panic disorder, resulting in significant social and vocational impairment, described significant reductions in symptoms following ingestion of psilocybin mushrooms.^20^ The patient reported as symptom free at 12-week follow-up, and continued to have sustained improvement for 18 months.^20^ Regrettably, case reports do not include precise doses, details of psychotherapy or validated blinded assessment measures. We are aware of only one open-label trial conducted in patients with OCD.^25^ Moreno et al^25^ enrolled nine participants who had an average of 3.4 previous medication treatment failures. Patients received escalating doses of psilocybin (7 mg/70 kg – 21 mg/70 kg), each separated by at least a week. In the 24-h post-administration monitoring period, patients experienced anything from a 23 to 100% reduction from baseline on the Yale–Brown Obsessive–Compulsive Scale (YBOCS), indicating a marked relief from symptoms.^25^ Two-thirds of patients maintained a greater than 50% decrease in YBOCS score at 24 h for at least one of the four single doses of psilocybin. Two patients reported symptomatic relief lasting nearly a week, with one patient still in remission at the 6-month follow-up.^25^

These preliminary reports are promising and serve as support for an early-phase clinical trial investigating the potential of PAP to treat OCD. However, prior trials did not follow an established and detailed clinical protocol. Here, we describe a comprehensive protocol for evaluating PAP as a treatment for treatment-resistant OCD, based on prior PAP interventions that have demonstrated efficacy in depression.^26^

## Method

### Overview

The protocol outlined below describes a pilot study assessing the feasibility, tolerability, safety and the clinical effects of PAP for treating patients with treatment-resistant OCD. This comprehensive study incorporates neurophysiology measures, including electroencephalogram, TMS and functional magnetic resonance imaging (fMRI), which will be used to examine the neural mechanisms associated with PAP, both during and after treatment. The results of this trial will inform the design and execution of a phase 2 randomised controlled trial.

### Primary objectives and hypotheses

The primary objective is to assess the feasibility, tolerability and safety of psilocybin, administered with psychological support, to adult participants with treatment-resistant OCD. Our hypothesis is that one session of psilocybin 25 mg administered under supportive conditions will be feasible (as measured by recruitment, drop-out and retention rates), tolerable (as measured by adverse events) and safe (as measured by serious adverse events).

Another objective is to assess the preliminary clinical effects of psilocybin, administered with psychological support, to adult participants with treatment-resistant OCD. Our hypothesis is that one session of psilocybin 25 mg administered under supportive conditions will be effective in reducing OCD symptoms at 1 week post-dosing.

### Exploratory objective

The exploratory objective is to compare neuroimaging and neurophysiological markers pre- and post-PAP in treatment-resistant OCD.

### Study design, setting and ethics

The trial will be a proof-of-concept, open-label, single-group design with ten participants. All study procedures will take place at the Centre for Addiction and Mental Health (CAMH), an academic hospital in Toronto, Canada. The authors assert that all procedures contributing to this work comply with the ethical standards of the relevant national and institutional committees on human experimentation and with the Helsinki Declaration of 1975, as revised in 2013. All procedures involving human patients were approved by from the CAMH research ethics board and a No Objection Letter from Health Canada; it has been registered on ClinicalTrials. Gov (identifier NTC06299319, registered on 15 February 2024). All staff on the study have undergone Good Clinical Practice training.

### Eligibility criteria

Participants included in the trial must have a primary diagnosis of OCD and meet criteria for treatment-resistance. Treatment resistance is defined as a failure to respond to two or more pharmacotherapy interventions (SSRIs or serotonin norepinephrine reuptake inhibitors (SNRIs)) at maximum dosage for at least 8 (for SSRI) or 12 (for SNRI) weeks of full course duration or discontinuation owing to intolerable side-effects, and one or more trials of a recognised psychological intervention with at least 12 sessions.^6,27^ Patients with OCD are often prescribed SSRIs and atypical antipsychotics, and these medications can attenuate the acute psychological effects of psychedelics. As such, participants will be required to taper off these concomitant medications over the screening period, to be eligible for the study. For a complete list of inclusion and exclusion criteria, along with lifestyle considerations for participants, please see Table 1. Additionally, there are eligibility criteria that must be met to act as a therapist in this study. Therapists involved in the trial must be licensed to provide therapy by a regulatory body. Unlicensed therapists will be directly supervised by a licensed therapist. Two study therapists trained in PAP will support the participant during the dosing session. There will be one therapist present at all times throughout the dosing session. The total treatment time will be 5–6 h, until after the acute effects of the psilocybin have passed.

**Table 1:** Inclusion and exclusion criteria

Inclusion criteria: Adults aged 18–65 years Are out-patients Must be deemed to have capacity to provide informed consent Must sign and date the informed consent form Stated willingness to comply with all study procedures Ability to read and communicate in English, such that their literacy and comprehension is sufficient for understanding the consent form and study questionnaires, as evaluated by study staff obtaining consent Primary DSM-5 diagnosis of OCD based on medical records and assessment using the SCID-5 administered at the first screening visit Participants diagnosed with treatment-resistant OCD defined as individuals with a score of ≥ 16 on the YBOCS (i.e. moderate symptom severity) and that have not responded to two or more separate pharmacological interventions and one or more trials of CBT; there is no upper limit on the number of treatment failures Individuals with an eGFR above 40 mL/min/1.73 m^2^ and all blood work within normal limits as assessed by clinical laboratory tests at screening (visit 1) Able to take oral medication Individuals who are capable of becoming pregnant: use of highly effective contraception for at least 1 month before screening and agreement to use such a method during study participation Individuals who are willing to and have tapered off current OCD medications for a minimum of 2 weeks before baseline (visit 2) and whose physician confirms that it is safe for them to do so Individuals who are willing to and have tapered off current inhibitors of 5’-diphospho-glucuronosyltransferase (UGT)1A9 and 1A10, aldehyde dehydrogenase inhibitors and alcohol dehydrogenase inhibitors for a minimum of 2 weeks (or more depending on the medication) before baseline (visit 2) and for the duration of the study, and whose physician confirms that it is safe for them to do so Agreement to adhere to lifestyle considerations (see below) throughout study duration Exclusion criteria: Pregnant as assessed by a urine pregnancy test at screening (visit 1) and baseline (visit 2) or individuals that intend to become pregnant during the study or are breastfeeding Treatment with another investigational drug or other intervention within 30 days of screening (visit 1) Have initiated psychotherapy in the preceding 12 weeks before screening (visit 1) Have a DSM-5 diagnosis of substance use disorder (use of tobacco and prescribed opioids are permitted) within the preceding 6 months Have active suicidal ideation as determined by the C-SSRS and/or clinical interview. Significant suicide risk is defined by suicidal ideation as endorsed by items 4 or 5 of the C-SSRS Any DSM-5 lifetime diagnosis of a schizophrenia spectrum disorder, psychotic disorder (unless substance induced or due to a medical condition), bipolar disorder type 1 or 2, paranoid personality disorder, borderline personality disorder or neurocognitive disorder as determined by medical history and the SCID-5 clinical interview Any first-degree relative with a diagnosis of schizophrenia spectrum disorder, psychotic disorder (unless substance-induced or due to a medical condition) or bipolar disorder type 1 or 2 as determined by the family medical history form and discussions with the participant Have contraindications to TMS as determined by the TASS questionnaire Have a history of seizures Are taking anticonvulsants or benzodiazepines (lorazepam up to 2 mg/day is acceptable) Presence of a relative or absolute contraindication to psilocybin, including a drug allergy, history of a stroke in the past year, uncontrolled hypertension, low blood pressure defined as ≤90/60 mmHg or labile blood pressure defined as recurrent, sudden, unexplainable blood pressure surges to levels of 140/90 mmHg or higher, history of myocardial infarction in the past year, cardiac arrhythmic, severe coronary artery disease, or moderate to severe renal or hepatic impairment, defined by an eGFR ≤40 mL/min/1.73 m^2^ for moderate renal impairment, and a total bilirubin of ≤50 *μ*mol/L and serum albumin ≤3.5 for moderate hepatic impairment (please note that participants must have an eGFR above 40 mL/min/1.73 m^2^ and within normal limits on the results in all clinical laboratory tests, including liver function tests at screening (visit 1), and please refer to the inclusion criterion 9 for details) Use of classic psychedelic drugs such as the serotonergic psychedelics drugs, psilocybin, ayahuasca, and 5-methoxy-N,N-dimethyltryptamine, etc. within the previous 12 months OR Any other clinically significant physical illness including chronic infectious diseases or any other major concurrent illness that, in the opinion of the investigator, may interfere with the interpretation of the study results or constitute a health risk for the participant if they take part in the study Lifestyle considerations: Abstain from alcohol for 24 h before the start of the psilocybin treatment session and for up to 6 h after administration Abstain from the use of any prescribed opioids, benzodiazepines or sleep aids (Z-drugs) within 12 h before the psilocybin treatment session and for up to 6 h after administration Abstain from any illicit drugs (e.g. cocaine, ecstasy/3,4-methylenedioxymethamphetamine, hallucinogens) and cannabis for the duration of the study. Presence of these substances will be assessed at a urine drug screening at visit 1 and baseline (visit 2) Abstain from driving or operating heavy machinery for up to 24 h after the psilocybin administration

OCD, obsessive–compulsive disorder; SCID-5, Structured Clinical Interview for DSM-5; YBOCS, Yale–Brown Obsessive–Compulsive Scale; eGRF, estimated glomerular filtration rate; C-SSRS, Columbia Suicide Severity Rating Scale; TMS, transcranial magnetic stimulation; TASS, Transcranial Magnetic Stimulation Adult Safety Screen.

### Research procedures

#### Recruitment

We will recruit via out-patient referrals from clinicians at CAMH and surrounding hospitals in the Greater Toronto Area. Regardless of recruitment method, all participants will be required to be under the care of a physician at CAMH to ensure clinical oversight; their usual treating psychiatrist will also be informed of their participation in the study. Participants who have expressed interest in the study will be contacted by the study team for a prescreening call where a description of the study will be given and a few short questions regarding psychiatric and medical history will be asked. The prescreen form will be reviewed by the principal investigator, and participants who are preliminarily deemed eligible will be contacted to schedule a consent and screening visit.

#### Informed consent and screening

Consent will be obtained in-person before the start of any research activities by trained study personnel. The principal investigator and/or therapists will not obtain informed consent. Each participant will be provided with a current copy of the research ethics board-approved informed consent form before the consent discussion. Research personnel will explain the clinical trial to the participant and answer any questions that may arise. Participants may take as much time as they need to make their decision, and may consult with others (e.g. family members, other healthcare providers, etc.) if they prefer.

Once consent is obtained, the screening process will begin. An outline of all assessments conducted during screening and throughout the study can be found in Table 2. The study physician will discuss tapering options with the patient based on the half-life of the medication and participant preference. Participants must be tapered off all concomitant medication 2 weeks before baseline to be eligible for the study.

**Table 2:** Overview of study activities and assessments

	Screening period		Intervention period					Primary end-point	Follow-up period			
Procedures	Screening (visit 1)	Washout period (3–6 weeks)	Baseline (visit 2, day −7 to day −1)	Pre-psilocybin neurophysiology (visit 3, day −7 to day −1)	Psilocybin session – dose (visit 4, day 0)	1 day post-intervention (visit 5, day 1)	Post-psilocybin neurophysiology (visit 6)	1 week post-intervention (visit 7, day 7)	3 weeks post-intervention (visit 8, day 21)	6 weeks post -intervention (visit 9, day 42)	9 weeks post-intervention (visit 10, day 63)	12 weeks post-intervention (visit 11, day 84)
Allowable window		Weekly		≤7 days from visit V4	≤7 days from baseline	None	≤7 days	±3 days	±3 days	±3 days	±3 days	±3 days
Informed consent	✔											
Demographics	✔											
Medical history	✔		✔									
Prior/concomitant medication review	✔	X------------------------------------------------------------------------------------X										
Inclusion/exclusion criteria review	✔	✔	✔									
MRI screening form	✔											
TASS	✔											
SCID-5	✔											
Vital signs (blood pressure, pulse)	✔		✔		✔							
Vital signs (body temperature)					✔							
Weight	✔											
Height	✔											
Electrocardiography	✔											
Clinical laboratory tests^14^	✔											
Urinalysis	✔											
Urine drug screening	✔		✔									
Urine pregnancy test^29^	✔		✔		✔							
Documentation of birth control	✔											
fMRI			✔						✔			
YBOCS	✔		✔			✔		✔	✔	✔	✔	✔
C-SSRS^30,31^	✔	✔	✔		✔	✔		✔	✔	✔	✔	✔
CGI^18^			✔			✔		✔	✔	✔	✔	✔
Neuronavigation				✔			✔					
Resting electroencephalogram				✔			✔					
Task electroencephalogram				✔			✔					
TMS-electroencephalogram				✔			✔					
Preparatory/integrative therapy and psychoeducation^5^			✔		✔	✔		✔				
120 min electroencephalogram					✔							
Psilocybin dose (25 mg)					✔							
Adverse event and serious adverse event review and evaluation	✔	✔	✔	✔	✔	✔	✔	✔	✔	✔	✔	✔
Source documentation and CRF collection	✔	X------------------------------------------------------------------------------------X										
MSI-BDP	✔											
5D-ASC^15^					✔							
MEQ^15^					✔							
GAD-7			✔			✔		✔	✔	✔	✔	✔
PHQ-9			✔			✔		✔	✔	✔	✔	✔
WEMWBS			✔			✔		✔	✔	✔	✔	✔
WHODAS 2.0			✔			✔		✔	✔	✔	✔	✔
WHO-QOL-BREF			✔			✔		✔	✔	✔	✔	✔

MRI, magnetic resonance imaging; TASS, Transcranial Magnetic Stimulation Adult Safety Screen; SCID-5, Structured Clinical Interview for DSM-5; fMRI, functional magnetic resonance imaging; YBOCS, Yale–-Brown Obsessive-–Compulsive Scale; C-SSRS, Columbia Suicide Severity Rating Scale; CGI, Clinical Global Impression scale; TMS, transcranial magnetic stimulation; CRF, case report form; MSI-BDP, Mclean Screening Instrument for Borderline Personality Disorder; 5D-ASC, 5-Dimensional Altered States of Consciousness scale; MEQ, Mystical Experiences Questionnaire; GAD-7, Generalised Anxiety Disorder-7; PHQ-9, Patient Health Questionnaire-9; WEMWBS, Warwick–Edinburgh Mental Wellbeing Scale; WHODAS 2.0, World Health Organization Disability Assessment Schedule 2.0; WHO-QOL-BREF, World Health Organization Quality of Life Questionnaire – Brief Version.

#### Study visits

**Fig. 1:**
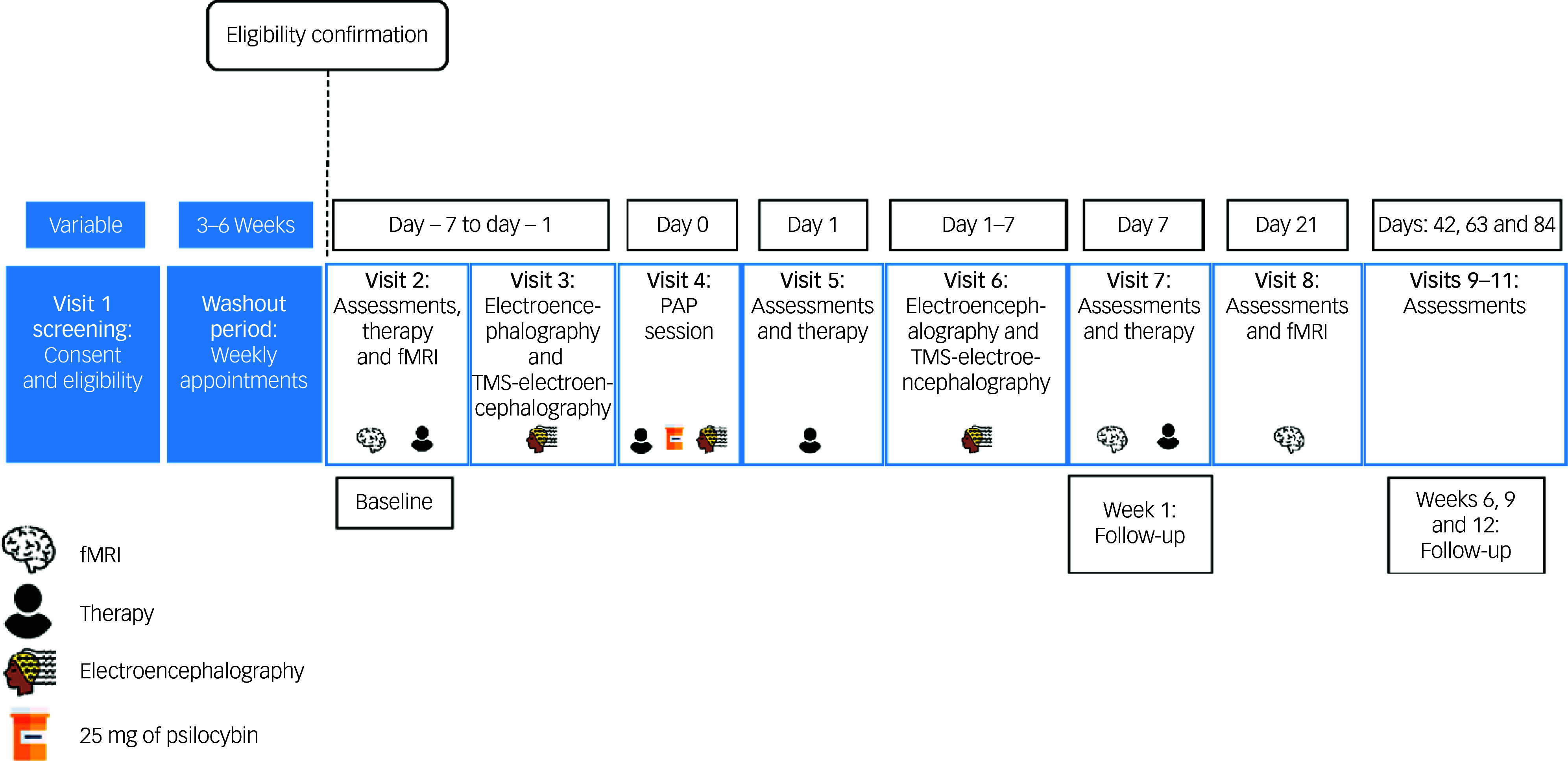
Outline of study visits. fMRI, functional magnetic resonance imaging; PAP, psilocybin-assisted psychotherapy; TMS, transcranial magnetic stimulation.

The outline of study visits is shown in Fig. 1 and an overview of all study activities and assessments can be found in Table 2. After the tapering period, participants will participate in a minimum of ten study visits over the period of 13 weeks. Additional study visits may be added at the discretion of the study physician or visits may be separated to reduce the burden on participants. Across these study visits, participants will take part in one psilocybin dosing session (25 mg) and two fMRI scans. Participants will also complete two TMS-electroencephalogram sessions, and three electroencephalogram recordings (one of which will occur during the dosing session). There will be six follow-up visits, which will occur in the final 12 weeks of the study, at which baseline assessments will be repeated.

#### Baseline, 1-week follow-up and 3-week follow-up

Baseline (visit 2) has similarities to both the 1-week and 3-week follow-up visit. All participants will undergo an fMRI scan at baseline and week 3. Electroencephalogram recordings (rest and task) and TMS-electroencephalogram will occur at baseline and 1–7 days post-dose, rather than at week 3. In addition, several assessments evaluating OCD symptom severity, anxiety, depression, well-being and quality of life, among others, will be administered at both time points. On the baseline day, preparatory therapy will be conducted, and the 1-week follow-up will include integration therapy for participants, both of which are outlined below.

#### Therapy

This study involves up to 6 h of manualised therapy administered before and after the dosing session, along with up to 8 h of supportive therapy during dosing. Therapy will be administered by two trained therapists who will be trained in PAP; therapists will have continunity with participants for the duration of the trial. The manual used for training is the Yale Manual for Psilocybin-assisted Therapy for OCD, delivered by a clinician who is an expert in OCD. Briefly, preparatory therapy will occur before dosing and will be used to answer participant questions, educate the participant regarding what to expect from the therapy, set boundaries and safety protocols for the dosing session and set intentions for the dosing session. On the dosing day, participants will lay on a bed with the option of using eyeshades. To enhance inward reflection, a pre-selected music playlist will be played quietly. All participants will listen to the same playlist, which is preset specifically for the standardisation of PAP sessions across all other psilocybin studies in our institution. Therapists will offer support and encourage inward reflection during the experience. At 1 day and 1 week after the dosing session, participants will participate in integration therapy, which is expected to last 1–3 h.

#### Dosing session

Participants will be administered a high dose of psilocybin (25 mg) in the form of a capsule. We chose this dose for standardisation across the study participants, as it is the most frequently chosen dose in other psilocybin research studies, with almost guaranteed and observed psychedelic effects.^32^ A recent study reported no significant differences in weight-adjusted dosing versus fixed dosing.^33^ Furthermore, advantages of fixed dosing outweigh the potential minimal advantage of weight-adjusted dosing, given that fixed dosing incurs a lower cost. Although the acute effects of orally ingested psilocybin typically last 4–6 h, participants will be expected to remain in the dosing suite for at least 5 h. There will be at least one therapist present at all times during the dosing session, and a medical professional will be on site to oversee medical care and approve patient discharge at the end of the day. In addition, participants will undergo an electroencephalogram recording, which will begin 5-min before drug administration and last for a total of 120 min. At the end of the dosing day, participants will be released to a caregiver, who will transport them home and remain with them for 24 h post-dose. For safety purposes, both the participant and caregiver will be provided with the contact information of a study team member who will be on-call should support be required.

#### Follow-up visits

Remote or in-person follow-up visits to assess changes on secondary outcome measures will occur at 1 day post-dose, as well as at 1, 3, 6, 9 and 12 weeks post-dose. These visits will be short, with the primary focus being the administration of the secondary assessment measures described below.

### Primary outcomes and measures

#### Feasibility outcomes

Feasibility will be assessed through recruitment and retention rates. Drop-out rates during three periods will be evaluated, namely (a) the screening period, in which the participant undergoes medication tapering and washout; (b) during the acute course of the study intervention and (c) during the follow-up period. A CONSORT diagram will be presented displaying participant retention rates throughout each phase of the trial, including recruitment. For full transparency, we will provide reasons for screening failures, withdrawals and drop-out.

#### Tolerability and safety outcomes

To evaluate the co-primary outcome of the safety of PAP in participants with treatment-resistant OCD, standardised adverse event monitoring will be conducted at all time points, starting from the first screening visit through to the final follow-up visit. During the dosing session, adverse events will be assessed both before and after the intervention, as well as the following morning when the participant returns for integration therapy. The Principal Investigator will closely monitor and assess all reported adverse events on the same day and will document them promptly in the adverse events log, to ensure the safety of all participants. The adverse event log is pre-approved by CAMH to be used in clinical trials. All adverse events will be assessed by the physician and Principal Investigator, and recorded in the log, detailing seriousness, unexpectedness, relatedness to the psilocybin, severity, actions taken with the psilocybin drug and any medications administered. This will ensure comprehensive documentation of the tolerability of psilocybin. Throughout the time periods mentioned above, we will prioritise the close and thorough evaluation of the acute and subacute psychological safety profile. The Columbia Suicide Severity Rating Scale (C-SSRS) will be administered at every study visit. The C-SSRS is a semi-structured interview designed to assess the severity and intensity of suicidal ideation, suicidal behaviour and non-suicidal injurious behaviour over the time period between study visits.^31^ There will be a psychiatrist on call to assess participants with active suicidal ideation, plans and/or intent, and standard of care will be provided. Adverse events will be classified based on severity, causality and expectedness by the study investigator. Serious adverse events are defined as any event that occurs which results in death, is life threatening, requires admission to hospital results in persistent or significant disability. All serious adverse events will be reported to the Principal Investigator, ethics committee, drug manufacturer and regulatory bodies within 24 h of the study team being made aware of the event, or according to the necessary reporting requirements of each department.

#### Clinical outcomes

The preliminary clinical effects of PAP will be measured according to change in the primary outcome measure, from baseline to week 1 total scores: the YBOCS will be employed. The YBOCS comprises ten items with total scores ranging from 0 to 40. Higher scores indicate greater symptom severity. The scale is clinician-rated, with interviewers providing a rating from 0 (‘no symptoms’) to 4 (‘extreme symptoms’) on each item.

### Additional exploratory outcomes and measures

#### Exploratory clinical effects outcomes

The clinical effects of PAP will also be investigated in the context of response and remission rates by comparing baseline to week 1 total scores on the YBOCS. Response is defined as a 35% reduction or more on the YBOCS.^34^ Remission is defined as as score of ≤7 on the YBOCS for patients with OCD.^34^ We will also analyse changes in YBOCS score from baseline to day 1 and weeks 3, 6, 9 and 12. In addition, baseline scores on various assessment measures to evaluate overall functioning, anxiety, depression, quality of life, disability and wellbeing will be compared with week 1. Anxiety will be measured with the Generalised Anxiety Disorder-7 (GAD-7) scale which is a brief self-report measure of generalised anxiety that has demonstrated good psychometric properties and is a widely used research instrument for assessing adult anxiety.^35^ The Patient Health Questionnaire-9 (PHQ-9) is a nine-item brief self-report questionnaire that will be employed to evaluate the presence and severity of depression.^36^ Quality of life will be evaluated with the World Health Organization Quality of Life Questionnaire – Short Version (WHO-QoL-BREF). The WHO-QoL-BREF is a self-report measure developed by the World Health Organization.^37^ It uses a five-point Likert scale to assess quality of life in the following areas: physical, psychological, level of independence, social relationships, environment and spirituality/religion/personal beliefs.^37^ The Disability Assessment Schedule (WHODAS 2.0) is a 12-item self-report questionnaire that assesses health status and disability across different cultures and settings.^38^ Participants will rate themselves on a Likert scale from 1 (‘none’) to 4 (‘extreme’) across six different domains of functioning: cognition, mobility, self-care, getting along, life activities and participation.^38^ The Clinical Global Impression scale (CGI) will also be used as a secondary outcome measure. The CGI is a brief observer-rated instrument that measures the clinician’s view of the patient’s global functioning before and after initiating the study medication.^39^ Well-being will be assessed withthe Warwick–Edinburgh Mental Wellbeing Scale (WMWBS), a self-report scale that consists of positively worded statements covering feelings and functioning aspects of mental well-being.^40^

### Exploratory outcomes and measures

#### Psychedelic experience

Potential response predictors, including the psychedelic experience itself, will be assessed as mediators/moderators of the primary outcome. The Five Dimension Altered States of Consciousness Questionnaire (5D-ASC) and the Mystical Experiences Questionnaire (MEQ) will be assessed for correlations with the primary and secondary outcomes as possible predictors of response. The 5D-ASC is a 94-item self-rated measure that consists of five subscales used to evaluate the psychedelic experience: (a) oceanic boundlessness, (b) anxious ego dissolution, (c) visionary re-structuralisation, (d) auditory alterations and (e) reduction of vigilance.^41,42^ The MEQ consists of a subset of 30 items from a larger scale that assesses four factors: mystical experiences (including ‘internal unity’, ‘external unity’, ‘noetic quality’ and ‘sacredness’), positive mood (awe, joy, peace and tranquillity), transcendence of time and space (sense of being outside of time, in a realm of no space boundaries, sense of timelessness) and ineffability (e.g. inability to adequately describe one’s experience in words).^29,43^

#### Neuroimaging

Participants will complete a 1 h MRI scan before and following PAP, including a T1 MRI, 28 min of resting-state fMRI, and a multi-shell diffusion MRI scan for exploratory analyses. Data will be collected with state-of-the-art MRI protocols developed as part of the Adolescent Brain Cognitive Development study, including hyperband sequences. MRI scans will undergo rigorous quality control checks, and will be cleaned using an extensive noise regression procedure, and normalised into surface-space with the Human-Connectome analysis tools. For resting fMRI, leading eigenvector dynamics analysis will be applied to examine dynamic functional connectivity, as was done in prior work on the effects of psilocybin in major depressive disorder.^44^ We will examine changes in dynamic connectivity measures related to specific ‘brain states’ from pre to post treatment. We hypothesise that we will see a greater repertoire of dynamic changes, associated with a reduction in overexpressed pathological brain states.

#### Neurophysiology

There are several exploratory neurophysiological measures in this study. The ongoing electroencephalogram recordings during the dosing session will be used to investigate changes of brain dynamics (spectral changes, changes in connectivity, changes in complexity measures) associated with psilocybin under acute conditions in patients with OCD. TMS-electroencephalogram measurements will be acquired as additional markers of the brain response to perturbation, targeting both the dorsomedial prefrontal cortex and dorsolateral prefrontal cortex. In addition, changes in resting-state electroencephalogram, task electroencephalogram and TMS-electroencephalogram between baseline, provocation of OCD symptoms and up to 1 week post-dose will also be used to investigate correlations between changes in electroencephalogram and TMS-electroencephalogram measures and changes in symptom severity.

During each of the two neurophysiology visits (one before and one after the intervention), resting-state electroencephalogram, task-related electroencephalogram and TMS-electroencephalogram (targeting two anatomical areas) will be collected, alternating between neutral and symptom provocation phases. OCD symptoms will be provoked by using an individualised external provocation method;^30,45^ For example, participants may be asked to touch a surface they perceive as unclean. The severity of OCD symptoms will be assessed during each phase by using an 11-point visual analogue scale (VAS). A provoked session will aim to elicit a VAS score between 4 and 7, as scores above 7 may cause significant distress, whereas scores below 4 may not sufficiently activate the brain regions associated with OCD symptoms. During the neutral phase, participants will engage in breathing exercises to relax and aim to bring their VAS score below 4. These two phases will then be repeated when targeting the other brain region. The duration of the electroencephalogram and TMS-electroencephalogram measurements will be approximately 80 min.

For the rest electroencephalogram, the participant will be seated comfortably with their eyes open, looking at a fixation target, for a period of 5 min. For task electroencephalogram, the participant will additionally perform an auditory oddball task consisting of 600 auditory stimuli (sinus tones, intensity of 80 dB, duration 40 ms) delivered binaurally through headphones, with an interstimulus interval of 1500 ms.^28^ The duration of this measurement will be about 15 min.

TMS-electroencephalogram will also be applied in blocks consisting of 150 biphasic single pulses of TMS with an inter-pulse interval ≥2.5 s, using masking noise to reduce the auditory-evoked electroencephalogram potential. Target stimulation sites will be left dorsomedial prefrontal cortex and left dorsolateral prefrontal cortex (the order will be counterbalanced between different study participants, but remain consistent in any given study participant). In total, the TMS-electroencephalogram procedure will take about 20 min.

Finally, functional connectivity change profiles associated with the neurobiological effects of PAP will be investigated by examining functional connections, using electroencephalogram at baseline compared with 1–7 days post-dose, and fMRI at baseline compared with week 3.

### Sample size calculation

Sample size was based on the primary outcome measure of YBOCS score. We based this on the primary outcomes from the study of Moreno et al,^25^ which established marked reductions in YBOCS at 24 h for different dose groups and presented effect sizes ≥1.2 (within group Cohen’s *d*).

Using an effect size of *d* = 1.2 and an alpha level of 0.05 for statistical significance will require *N* = 10 completers to achieve 90% power. This calculation is for a single-arm design (two-sided), using a paired-sample *t*-test. This sample size will be able to detect large correlations (*r* = 0.71) between change scores, with an alpha of 0.05 and 80% power.

### Statistical methods and data management

Characteristics of the trial cohort will be summarised by mean (s.d.) and median (minimum, maximum) values. Summary raw scores will be presented at each assessment time both numerically and graphically. The small sample size means that conservative nonparametric testing is required to address the primary and secondary objectives. Exact paired permutation t-tests will be used to determine whether PAP achieves a 35% reduction in YBOCS symptoms.

### Dissemination

The results of this study will be used to support a funding bid for larger, confirmatory randomised controlled trials. The results will also be published in academic journals and presented in academic domains, including scientific conferences.

## Discussion

There has been a recent resurgence in exploration of the therapeutic potential of PAP for several mental disorders. Case reports and results from one preliminary study have demonstrated a potential therapeutic benefit of PAP for the treatment of OCD. However, there are currently no recent published data exploring the effects and safety of PAP in these populations.

This protocol will serve as a proof-of-concept trial evaluating the safety, feasibility and preliminary clinical effects of PAP in adults with OCD. Data from this trial will be used to inform the development of larger-scale randomised controlled trials. The pilot trial also provided important neurobiological assessments (MRI, electroencephalogram and TMS-electroencephalogram), which can inform future mechanistic trials. We anticipate that PAP will be safe and provide some therapeutic benefit through increasing cognitive flexibility and ‘resetting’ maladaptive networks. Furthermore, the protocol outlined is not limited to OCD, and could be adapted to other clinical populations such as body dysmorphic disorder.^46^ According to the preliminary results from an early case report and an open-label trial of single-dose psilocybin for body dysmorphic disorder, as a related disorder to OCD, a reduction in obsessions and compulsions on the body dysmorphic disorder version of the YBOCS was observed.^47,48^ In addition, this study reported that early changes in resting-state brain connectivity, including an increase in connectivity between nodes in the executive control network and the DMN, predicted response after 1 week.^49^

As the field of psychedelic research expands, transparency and standardisation in clinical trials will help facilitate research and ensure best practices are being followed across studies.

The result of this clinical trial will inform the safety and tolerability of using 25 mg of psilocybin as a treatment for adults with OCD, and help to provide a standardised procedure for future psychedelic research in clinical trials.

## Data Availability

Data availability is not applicable to this article as no new data were created or analysed in this study.
